# Exploring how levetiracetam mitigates toxicity and ameliorates memory impairment

**DOI:** 10.3389/fphar.2025.1651414

**Published:** 2025-09-11

**Authors:** Ahmad H. Alhowail, Abeer M. Alharbi

**Affiliations:** Department of Pharmacology and Toxicology, College of Pharmacy, Qassim University, Buraydah, Saudi Arabia

**Keywords:** levetiracetam, chemotherapy, inflammation, oxidative stress, synaptic plasticity

## Abstract

Cognitive impairment encompasses a spectrum of deficits that markedly affect daily functioning and quality of life. Understanding the specific cognitive domains involved is thus crucial for developing targeted interventions and effective support strategies. This impairment ranges from mild cognitive decline to severe dementia and disproportionately affects older adults and cancer survivors. Multiple pathophysiological mechanisms, including elevated neuroinflammation, oxidative stress, disrupted synaptic plasticity, and neuronal apoptosis, contribute to the onset and progression of cognitive dysfunction. Emerging clinical and experimental data suggest that pharmacological interventions, including levetiracetam (LEV), a second-generation antiepileptic drug, can attenuate cognitive impairment. The neuroprotective potential of LEV is attributed to its unique mechanism of action, which involves selective binding to synaptic vesicle protein 2A and modulation of neurotransmitter release. In addition to its well-established antiepileptic effects, LEV exhibits anti-inflammatory and antioxidant properties, suggesting broader therapeutic applications in mitigating cognitive decline. This review synthesizes current knowledge on the mechanisms underlying cognitive impairment, evaluates existing measurement and prevention approaches, along with their limitations, and critically examines the potential efficacy of LEV in this context. The novelty of this review lies in its integrative focus on the mechanistic pathways through which LEV may protect against cognitive decline, with attention to conflicting findings and unresolved questions. In conclusion, current evidence suggests that LEV is a promising therapeutic candidate beyond epilepsy, though further clinical studies are needed to confirm its efficacy.

## 1 Introduction

Cognitive impairment, ranging from mild cognitive decline to severe dementia, represents a growing global health concern, particularly among aging populations and cancer survivors (Hale et al., 2020). While dementia profoundly compromises daily functioning, mild cognitive impairment (MCI) is characterized by measurable deficits in cognition, while independence in daily activities is largely preserved (Jessen et al., 2020). In oncology, increasing evidence suggests that neurocognitive deficits arise not only as adverse effects of chemotherapy and radiotherapy but also as a consequence of cancer-related biological processes (Olson and Marks, 2019). Neuroinflammation and oxidative stress are now recognized as key contributors to cognitive dysfunction (d’Avila et al., 2018). These processes, mediated by cytokine dysregulation, reactive oxygen species (ROS) generation, and glial activation, can induce neuronal apoptosis and synaptic degeneration (Ahmad et al., 2022).

Levetiracetam (LEV) is a second-generation antiepileptic drug belonging to the pyrrolidone class. It is distinguished by its unique mechanism of action and favorable pharmacokinetic profile (Wright et al., 2013). Unlike traditional antiepileptic drugs, LEV selectively binds to synaptic vesicle protein 2A (SV2A), regulating neurotransmitter release and suppressing neuronal hyperexcitability (Contreras-García et al., 2022; Howard et al., 2018). In addition, it inhibits N-type calcium (Ca^2+^) and potassium (K^+^) channels and reduces intracellular Ca^2+^ release (Zwierzyńska and Pietrzak, 2022). Initially approved for epilepsy treatment, LEV has demonstrated efficacy in various seizure types, including focal, generalized, and neonatal seizures (Alfaro-Rodríguez et al., 2016), and is recommended as a first-line therapy, particularly for women of childbearing age and elderly patients, owing to its safety profile and low potential for drug–drug interactions (Celdran de Castro et al., 2023).

Beyond its established anticonvulsant role, LEV exhibits antioxidant, anti-inflammatory, and antiapoptotic properties, making it a promising neuroprotective agent in conditions such as traumatic brain injury, ischemic injury, and neurodegenerative diseases (Contreras-García et al., 2022; Zwierzyńska and Pietrzak, 2022). However, while preclinical evidence is encouraging, clinical findings remain mixed, and some studies report cognitive side effects in epilepsy patients, including irritability and memory complaints. This duality underscores the need for a cautious and critical appraisal of LEV’s broader applications (Sen et al., 2024).

**FIGURE 1:**
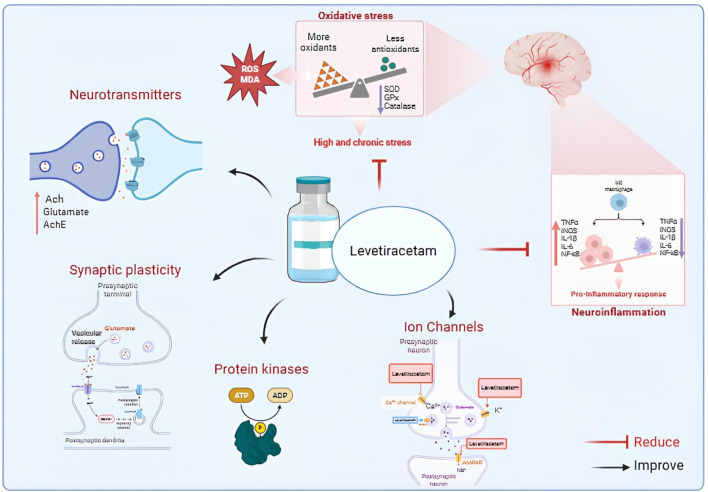
Proposed mechanisms of Levetiracetam action in neuronal function and neuroprotection. Levetiracetam (LEV) modulates neurotransmitters, synaptic plasticity, and ion channels, while reducing oxidative stress and pro-inflammatory responses. These combined effects improve neuronal stability and contribute to neuroprotection. Abbreviations: Ach, acetylcholine; AchE, acetylcholinesterase; ROS, reactive oxygen species; MDA, malondialdehyde; SOD, superoxide dismutase; GPx, glutathione peroxidase; TNF-α, tumor necrosis factor alpha; IL, interleukin; NF-κB, nuclear factor kappa B; iNOS, inducible nitric oxide synthase.

Given the rising prevalence of cognitive impairment among older adults and cancer survivors, and the multifactorial mechanisms underlying its pathogenesis—including chronic inflammation and oxidative stress (Bagnall-Moreau et al., 2019)—the pleiotropic actions of LEV make it an attractive candidate for neuroprotective interventions (Ewens and Thayer, 2025). In this review, we consolidate experimental evidence to highlight the ability of LEV to counteract cognitive decline, critically evaluating both positive and negative findings, and examining the underlying mechanisms across various domains: oxidative stress, neuroinflammation, neurotransmission, synaptic plasticity, and ion channel modulation (Figure 1). The objective is to provide a comprehensive framework to guide future research and therapeutic development.

However, it is important to acknowledge that the pathophysiology of cognitive impairment is highly heterogeneous, and not all mechanistic pathways implicated in preclinical models have been validated in humans. Moreover, the evidence for LEV outside epilepsy remains preliminary, with some studies reporting minimal or no cognitive benefit. This review therefore aims not only to summarize the literature but also to critically evaluate inconsistencies, gaps, and translational challenges.

### 1.1 Search strategy

A comprehensive literature search of the Medline, PubMed, Embase, Scopus, and Web of Science databases was conducted to identify studies examining the effects of LEV on cognitive impairment. The search targeted publications from 2004 to 2025 and included pertinent human and animal studies. Search terms included combinations of “LEV,” “animal,” “human” OR “behavior,” “animal/physiology” OR “LEV improve cognitive impairment/dysfunction.” Search results were meticulously filtered by examining titles and abstracts to ensure inclusion of only those study types, including experimental studies, RCTs, observational studies, and case reports, and studies excluded, such as editorials, letters, protocols, and conference abstracts. The investigation produced 96 published study records that were utilized in the current review article.

## 2 LEV and neuroinflammation

Neuroinflammation is the response of the central nervous system to infection, injury, or harmful stimuli (Oronsky et al., 2022), primarily mediated by microglia and astrocytes (Chen et al., 2017). While acute neuroinflammation can be protective, chronic activation leads to sustained immune responses, neuronal damage, and functional decline (Müller et al., 2025). Persistent neuroinflammation, marked by elevated proinflammatory cytokines such as interleukin (IL)-6, IL-1β, and tumor necrosis factor-alpha (TNF-α), is strongly implicated in synaptic dysfunction, neurotransmitter dysregulation, and cognitive decline (Bersano et al., 2023; DiSabato et al., 2016). Such elevations have been reported in Alzheimer’s disease, Parkinson’s disease, epilepsy, multiple sclerosis, traumatic brain injury, and chemotherapy-related cognitive impairment (“Neuroinflammation as a Link in Parkinson’s and Alzheimer’s Diseases: A Systematic Review and Meta-Analysis,” 2024; Stephenson et al., 2018; Zhang W. et al., 2023).

Microglial overactivation and the resulting cytokine surge disrupt synaptic plasticity and promote neuronal apoptosis, contributing to deficits in learning and memory (Zhao et al., 2019). For example, Zhao et al. found that chemotherapy-induced increases in IL-1β, TNF-α, and IL-4 levels were significantly correlated with the severity of cognitive decline in patients with breast cancer, underscoring the role of systemic inflammation in chemotherapy-related cognitive impairment (Zhao et al., 2019).

According to recent studies, LEV seems to exert neuroprotective effects by attenuating neuroinflammation and improving cognitive outcomes (Zhao et al., 2019). Further, LEV has been observed to markedly decrease IL-1β and TNF-α expression, mitigating neuronal damage and alleviating central inflammatory responses (Marini et al., 2004; Yao et al., 2021; Zou et al., 2013). Furthermore, LEV significantly suppresses nuclear factor kappa B (NF-κB) expression, a transcriptional activator of TNF-α, in a dose-dependent manner, suggesting that its anti-inflammatory effects are mediated via transcriptional regulation of cytokine expression (Mani et al., 2022). However, some chronic neurodegeneration models report no significant anti-inflammatory benefit, raising questions about the disease-specific contexts in which LEV is effective.

LEV has also been shown to improve systemic and cerebral metabolic profiles; in hyperglycemic states, it reduces IL-1β, TNF-α, and IL-6 levels and increases IL-10 levels in the brain (Zhang Y. -Y. et al., 2023). Furthermore, in models of epileptogenesis following status epilepticus, LEV has been found to inhibit the activation of BV-2 microglia, thereby limiting inflammation-associated neurodegeneration (Itoh et al., 2019).

Despite promising findings, not all studies demonstrate reductions in cytokines or improvements in cognition with LEV. Many preclinical models rely on acute injury paradigms that may not reflect the chronic, multifactorial nature of human cognitive decline. Future work should clarify whether observed cytokine changes consistently mediate cognitive benefit, and whether dose and timing of administration influence outcomes.

Altogether, these findings demonstrate that LEV mitigates neuroinflammation by modulating cytokine production and inhibiting inflammatory transcriptional pathways. Table 1 summarizes key experimental evidence.

**TABLE 1:** Anti-inflammatory actions of LEV in preclinical models.

Author(s)/Year	Model	Key findings
Marini et al. (2004)	Kainic acid-induced seizures	↓ IL-1β, ↓ neuronal damage
Yao et al. (2021)	Ischemic stroke	↓ IL-1β, ↓ TNF-α
Zou et al. (2013)	Traumatic brain injury	Reversed chronic IL-1β elevation
Mani et al. (2022)	Doxorubicin-induced rats	↓ TNF-α, ↓ NF-κB expression (dose-dependent)
Zhang Y. -Y. et al. (2023)	Streptozotocin-induced diabetic model	↓ TNF-α, ↓ IL-1β, ↓ IL-6, ↑ IL-10, ↓ glucose, suppressed microglial activation via JNK/MAPK/NF-κB pathway
Itoh et al. (2019)	Pilocarpine-induced status epilepticus	Suppressed microglial activation Pilocarpine: ↑ TNF-α, ↑ IL-1β LEV: ↓ TNF-α, ↓ IL-1β

## 3 LEV and oxidative stress

Oxidative stress is a critical driver of neurodegeneration, contributing to neuronal dysfunction and cognitive decline (Houldsworth, 2024). ROS generated from impaired mitochondrial function trigger inflammation and apoptotic pathways in key memory-related regions such as the hippocampus (Palma et al., 2023; Rauf et al., 2023). Excess ROS overwhelms antioxidant defenses, leading to lipid peroxidation, membrane damage, mitochondrial dysfunction, and impaired synaptic transmission (D’Alessandro et al., 2025; Guo et al., 2017).

Under physiological conditions, superoxide anions are converted to hydrogen peroxide by superoxide dismutase (SOD) (Andrés et al., 2023; Ighodaro and Akinloye, 2019), which is subsequently reduced to water by glutathione peroxidase and catalase (Afzal et al., 2023; Asatiani et al., 2025). Deficiencies in these antioxidants, or excessive ROS and malondialdehyde (MDA) accumulation, signify oxidative stress and are linked to cognitive impairment (Afzal et al., 2023). In the aging brain, hippocampal neurons are particularly susceptible to oxidative injury, resulting in reduced synaptic plasticity and learning capacity (Ionescu-Tucker and Cotman, 2021). Enhancing SOD and catalase activity has been reported to restore redox balance and improve cognitive function (Cram, 2022).

LEV exhibits potent antioxidant activity, significantly reducing hydrogen peroxide and lipid peroxidation levels (Haider et al., 2020). In addition, it increases SOD and catalase activities (Kataria et al., 2024). At the molecular level, LEV modulates oxidative stress by influencing the expression of solute carrier family 7 member 11 (also known as xCT) and inducible nitric oxide synthase, promoting cystine uptake for glutathione (GSH) synthesis and reducing basal glutamate levels (Oliveira et al., 2007). *In vivo*, LEV preserves hippocampal GSH levels, maintains catalase activity, and decreases lipid peroxidation and nitrite–nitrate accumulation (Marini et al., 2004; Oliveira et al., 2007). It has also been reported that LEV increases SOD and glutathione peroxidase activities and reduces MDA levels (Komur et al., 2014), although effects on non-enzymatic GSH levels are inconsistent (Mani and Rashed Almutairi, 2023). It should be noted that antioxidant effects of LEV are inconsistently reported across studies, with variable results for markers such as GSH and MDA. Furthermore, few studies directly link biochemical changes to measurable improvements in cognition, leaving uncertainty about causal pathways. Standardized assays and replication across independent laboratories are needed to validate these findings.

Collectively, these findings indicate that LEV exerts neuroprotective effects beyond seizure suppression, acting through antioxidant mechanisms to preserve neuronal function. Table 2 provides a summary of experimental models and outcomes.

**TABLE 2:** Antioxidant effects of LEV on oxidative stress pathways.

Author(s)/Year	Model	Key findings
Kataria et al. (2024)	Cell-based assay	↑ SOD, ↑ catalase
Oliveira et al. (2007)	Pilocarpine-induced seizures	↑ GSH, ↑ catalase, ↓ MDA, ↓ nitrites
Marini et al. (2004)	Kainic acid-induced toxicity	↓ MDA, ↑ GSH
Mani and Rashed Almutairi (2023)	LPS-induced cognitive deficits	↓ MDA, ↑ catalase, non-significant effect on GSH

## 4 LEV and neurotransmitter regulation

Neurotransmitters are key chemical messengers that facilitate signal transmission across synapses, enabling neuronal communication within the central nervous system (Teleanu et al., 2022). They regulate diverse physiological processes, including mood, sleep, memory, and motor control (Teleanu et al., 2022). In the context of cognition, they are vital for both short- and long-term memory formation and retrieval (Hansen et al., 2022). Disruptions in neurotransmitter homeostasis can impair cognitive performance (Feld and Born, 2019).

A balanced interplay between excitatory and inhibitory neurotransmission is fundamental for maintaining cognitive function and neuronal stability (Tatti et al., 2017). Dysregulation—particularly involving gamma-aminobutyric acid (GABA), glutamate, and acetylcholine (ACh)—is implicated in several neurological disorders and toxic insults leading to cognitive impairment (Kim and Yoon, 2017). GABA serves as the principal inhibitory neurotransmitter in the brain, whereas glutamate and ACh are major excitatory transmitters critical for memory processing, synaptic plasticity, and attention (Contreras-García et al., 2022; Haam and Yakel, 2017; Zhou and Danbolt, 2014).

Alterations in these systems are observed in epilepsy, Alzheimer’s disease, and chemotherapy-related cognitive impairment (Zhang et al., 2022). Notably, treatment with chemotherapeutic agents such as doxorubicin, cisplatin, and combination regimens of cyclophosphamide, methotrexate, and fluorouracil has been associated with elevated glutamate and dopamine levels, contributing to cognitive decline (Alhowail, 2025; Alhowail and Aldubayan, 2023; Alhowail et al., 2022). These changes are believed to result from increased extrasynaptic neurotransmitter pressure and membrane damage driven by heightened neuroinflammation and oxidative stress (Park et al., 2024).

LEV exerts modulatory effects on inhibitory and excitatory neurotransmission, contributing to synaptic stabilization (Hadera et al., 2025; Meehan et al., 2012). It reduces extracellular glutamate levels, protects against hippocampal injury, and preserves neural volume, thereby attenuating excitotoxicity and neuronal loss (Santana-Gómez et al., 2018). Furthermore, LEV indirectly enhances GABAergic neurotransmission by influencing GABA metabolism and counteracting the effects of negative allosteric modulators on GABA_*A*_ receptors, thereby stabilizing neuronal excitability (Micov et al., 2010). In parallel, it upregulates the expression of astrocytic glutamate transporters EAAT1 (GLAST) and EAAT2 (GLT-1), improving glutamate clearance and limiting excitatory overstimulation (Zou et al., 2013).

At the synaptic level, LEV restores impaired glutamate release and normalizes the paired-pulse facilitation ratio at CA3–CA1 hippocampal synapses, indicating improved responsiveness and reduced hyperexcitability (Salaka et al., 2022). It also appears to modulate N-methyl-D-aspartate (NMDA) receptor sensitivity and influence excitatory signaling via protein kinase C (PKC) and calcium/calmodulin-dependent protein kinase II (CaMKII), both of which are critical regulators of synaptic plasticity and memory encoding (Wang et al., 2019). In the cholinergic system, LEV restores ACh levels in a dose-dependent manner, particularly under inflammatory or toxic stress conditions (Mani and Rashed Almutairi, 2023). While LEV appears to reduce glutamatergic excitotoxicity and enhance GABAergic tone, excessive suppression of excitatory signaling could potentially impair aspects of learning and memory. Evidence in cholinergic modulation is similarly limited to small studies, with variable effects depending on species and experimental conditions. Comparative analyses with other neurotransmitter-targeting agents would help clarify whether LEV offers distinct or superior benefits.

These findings demonstrate the multifaceted role of LEV in modulating neurotransmitter systems central to excitability, synaptic transmission, and cognitive function. Key evidence is summarized in Table 3.

**TABLE 3:** Modulation of neurotransmitter systems by LEV.

Author(s)/Year	Model	Key findings
Santana-Gómez et al. (2018)	Lithium–pilocarpine–induced status epilepticus	↓ Extracellular glutamate, ↑ neural volume
Zou et al. (2013)	Traumatic brain injury	↑ EAAT1/EAAT2 expression, ↓ excitotoxicity
Salaka et al. (2022)	Epileptic (lithium–pilocarpine) rats	Restored paired-pulse facilitation, ↓ synaptic hyperexcitability
Mani et al. (2022)	LPS-induced toxicity in rats	↑ ACh (dose-dependent)
Mani et al. (2022)	Doxorubicin-induced toxicity in rats	↑ ACh, ↓ acetylcholinesterase activity
Alhowail et al. (2022)	Cyclophosphamide–methotrexate–fluorouracil-induced neurotoxicity	↑ Glutamate, ↑ dopamine
Alhowail et al. (2022)	Doxorubicin-induced neurotoxicity	↑ Glutamate receptor expression (AMPA/NMDA) ↑ NF-κB, TNF-α, cyclooxygenase-2, MDA, caspase-3, Bax ↓ SOD, catalase, GSH
Park et al. (2024)	Cisplatin-induced neurotoxicity	↑ TNF-α, IL-6, IL-1β, caspase-3, Bax ↓ IL-10, synapsin-1, synapsin-2, SOD, Bcl-2
Alhowail (2025)	Cisplatin-induced neurotoxicity	↑ Glutamate receptor expression (AMPA/NMDA) ↑ NF-κB, TNF-α, IL-6, ROS, Nrf-2, SOD, lipid peroxidation ↓ Mitochondrial complex I

## 5 LEV and synaptic plasticity

Synaptic plasticity refers to the capacity of synapses to strengthen or weaken over time in response to patterns of neuronal activity (Hansen et al., 2022). It underpins learning and memory consolidation (Murai and Goto, 2025). Long-term potentiation is a well-characterized form of synaptic strengthening following high-frequency stimulation (Spinelli et al., 2017), involving enhanced presynaptic neurotransmitter release and increased postsynaptic receptor sensitivity and density (Bhattacharya et al., 2020). Conversely, long-term depression represents synaptic weakening induced by low-frequency stimulation, which reduces neurotransmitter release or promotes postsynaptic receptor endocytosis (Cuntz et al., 2020).

LEV has been found to restore synaptic function and cognitive performance in preclinical models of neurodegeneration (Lin et al., 2023). It improves hippocampal structure and behavioral outcomes by reducing aberrant network activity and enhancing memory functions (Manchon et al., 2016; Sanchez et al., 2012). In models of brain trauma and seizure-associated injury, LEV partially reverses synaptic plasticity deficits, suggesting potential to promote neural circuit recovery (Chen et al., 2018).

In chronic epilepsy, LEV not only reduces seizure frequency but also enhances synaptic transmission and structural remodeling (Ewens and Thayer, 2025; Zheng et al., 2022). It restores basal synaptic responsiveness and paired-pulse facilitation in hippocampal circuits, reverses CA1 region atrophy, and improves cognitive performance (Salaka et al., 2022). Mechanistically, LEV has been reported to improve spatial learning and memory, augment field excitatory postsynaptic potentials, and upregulate expression of synaptic proteins such as postsynaptic density protein 95 (PSD-95) and neural cell adhesion molecule (NCAM) (Magalhães et al., 2015; Wang et al., 2019). It also normalizes growth-associated protein 43 levels, indicative of enhanced synaptic integrity and remodeling (Wang et al., 2019). Furthermore, LEV exhibits beneficial effects in systemic disease models that impair cognition (Mohamadi et al., 2025; Sanchez et al., 2012).

LEV interacts with intracellular kinase pathways essential for synaptic regulation and cognitive processing. It has been reported to inhibit β-site amyloid precursor protein cleaving enzyme 1 and presenilin-1, both of which are involved in early-onset Alzheimer’s disease pathogenesis (Zheng et al., 2022). Further, LEV administration has been shown to restore PKC and CaMKII activity, which are reduced following subthreshold convulsant discharge, and enhance expression of PSD-95 and NCAM, both of which are critical for synaptic plasticity and memory formation (Wang et al., 2019). In a model of bilateral common carotid artery stenosis (BCAS), LEV was reported to improve cognitive performance by increasing protein kinase A (PKA) and phosphorylated cAMP response element-binding protein levels, which are otherwise reduced in BCAS-induced cognitive impairment (Inaba et al., 2019) (Table 5).

The link between LEV and kinase activity is suggestive but largely correlative. Causal evidence—such as kinase inhibition or knockdown experiments—remains sparse, making it unclear whether these pathways are necessary for LEV’s cognitive effects. Additionally, reports of LEV modulating β-secretase and presenilin pathways in Alzheimer’s models require replication before strong conclusions can be drawn. Although improvements in LTP and synaptic protein expression have been observed, not all studies demonstrate parallel gains in behavioral cognition, raising questions about functional relevance. Moreover, most findings are from short-term experiments; durability of synaptic changes after treatment discontinuation remains largely unexplored. Including both positive and neutral results will provide a more balanced assessment.

Collectively, these findings support the role of LEV in preserving and restoring synaptic plasticity, a core mechanism underlying cognitive resilience. Supporting evidence is summarized in Table 4.

**TABLE 4:** Effects of LEV on synaptic plasticity and cognitive recovery.

Author(s)/Year	Model	Key findings
Manchon et al. (2016)	Neuronal cultures treated with doxorubicin	↓ γH2A.X levels, improved synaptic and cognitive function
Sanchez et al. (2012)	hAPPJ20 transgenic mice (Alzheimer’s disease model)	↓ Network hyperactivity, ↑ memory performance, ↑ hippocampal integrity
Chen et al. (2017)	Traumatic brain injury with seizures (kainic acid)	Partial reversal of long-term synaptic plasticity suppression
Salaka et al. (2022)	Epileptic rats (lithium–pilocarpine model)	↑ Paired-pulse facilitation, ↓ CA1 atrophy, ↑ cognitive function
Wang et al. (2019)	Subthreshold convulsant discharge model	↑ PSD-95, ↑ NCAM, increased growth-associated protein 43, ↑ memory and field excitatory postsynaptic potential slope
Wang et al. (2019)	Seizures	↑ PKC, ↑ CaMKII ↓ PSD-95, ↓ NCAM
Inaba et al. (2019)	BCAS	↑ PKA, ↑ phosphorylated cAMP response element-binding protein

## 6 LEV and ion channels

Ion channels are transmembrane proteins that facilitate the selective movement of ions, such as Ca^2+^, Na^+^, K^+^, and Cl^−^, across neuronal membranes (Kulbacka et al., 2017). They are crucial for maintaining the resting membrane potential, generating action potentials, and supporting synaptic transmission and plasticity (Burke and Bender, 2019; Debanne and Russier, 2019). Ion channel dysfunction is linked to cognitive decline in Alzheimer’s disease, Parkinson’s disease, epilepsy, and age-related cognitive impairment (Bhoi et al., 2025; Reddy and Estes, 2016).

Disruption of glutamate receptor function, specifically AMPA or NMDA receptors, can impair cognitive function and long-term potentiation (de León-López et al., 2025; Sumi and Harada, 2020). Overactivation of Ca^2^-permeable channels, including NMDA and voltage-gated Ca^2+^ channels, leads to excessive Ca^2+^ influx, triggering excitotoxicity and neuronal damage (Antunes et al., 2022; Matuz-Mares et al., 2022). Similarly, dysfunction of voltage-gated Ca^2+^, Na^+^, or K^+^ channels can impair neural circuits responsible for attention, working memory, and decision making (Barbieri et al., 2023; Wang et al., 2025).

LEV exerts part of its neuropharmacological effects through Ca^2+^ channel modulation. It inhibits CaV2.2 (N-type) Ca^2+^ currents in sympathetic neurons, reducing Ca^2+^ influx and synaptic transmission (Vogl et al., 2012). This effect is stereospecific, dependent on SV2A, and occurs via an intracellular mechanism (Vogl et al., 2012).

LEV also enhances renal outer medullary potassium channel 1 (ROMK1) channel activity via a PKA-mediated phosphorylation pathway (Lee et al., 2008), stabilizing the open state of this channel and restoring neuronal resting potential (Lee et al., 2008). Furthermore, LEV induces membrane hyperpolarization and reduces action potential firing in sensory neurons by concurrently activating K^+^ channels and inhibiting depolarization-triggered Ca^2+^ influx (Ozcan and Ayar, 2012).

In addition, LEV reduces the frequency of AMPA receptor-mediated events without altering their amplitude (Lee et al., 2009), suggesting selective modulation of receptor kinetics. Altogether, these actions may reduce excitotoxicity and neuroinflammation, thereby preserving neuronal integrity. While LEV clearly modulates N-type Ca^2+^ channels, results are not uniform across all preparations, and the degree of SV2A-dependence remains debated. Furthermore, broad suppression of calcium influx could theoretically dampen learning processes, highlighting a potential trade-off between excitotoxicity prevention and cognitive performance. These mechanistic uncertainties warrant careful investigation in translational studies. Supporting evidence is summarized in Table 5.

**TABLE 5:** LEV-mediated modulation of ion channels.

Author(s)/Year	Model	Key findings
Vogl et al. (2012)	Neuronal cultures	↓ Ca^2+^ influx, ↓ synaptic transmission, SV2A- and stereospecific inhibition
Lee et al. (2008)	*Xenopus* oocytes	↑ ROMK1 activity via PKA pathway, stabilized resting potential
Ozcan and Ayar (2012)	Dorsal root ganglion neurons in primary culture	↑ Membrane hyperpolarization, ↓ action potential firing, ↓ Ca^2+^ influx
Lee et al. (2009)	Male rats	↓ Frequency of AMPA receptor events, amplitude unchanged

## 7 Conclusion

In addition to its established antiepileptic effects, LEV exhibits promising neuroprotective properties. Through its anti-inflammatory, antioxidant, neurotransmitter-modulating, and synaptic-stabilizing actions, LEV has demonstrated potential to alleviate cognitive impairment associated with neurodegenerative diseases, brain injury, and chemotherapy-related cognitive decline. Evidence in both preclinical and clinical studies suggests that LEV regulates ion channel activity, enhances synaptic plasticity, and mitigates excitotoxicity. These findings underscore the potential of LEV as a therapeutic candidate for cognitive dysfunction. However, further controlled clinical trials need to be conducted to confirm its efficacy, define optimal dosing regimens, and establish long-term safety profiles. Taken together, the evidence for LEV as a cognitive protectant remains promising but inconclusive. Conflicting results, small sample sizes, and short follow-up periods limit the strength of current conclusions. Future randomized trials with standardized cognitive endpoints, mechanistic biomarkers, and long-term safety monitoring are essential before LEV can be recommended as a routine therapy for cognitive impairment.
